# Different platforms, different plots? The Kremlin-controlled search engine Yandex as a resource for Russia’s informational influence in Belarus during the COVID-19 pandemic

**DOI:** 10.1177/14648849231157845

**Published:** 2023-04-25

**Authors:** Daria Kravets, Anna Ryzhova, Florian Toepfl, Arista Beseler

**Affiliations:** 26580 University of Passau, Germany

**Keywords:** Search engines, conspiracy theories, Yandex, Google, Russia, foreign influence

## Abstract

Extant research demonstrated that the algorithms of the Kremlin-controlled search engine Yandex, compared to those of its US-based counterpart Google, frequently produce results that are biased toward the interests of Russia’s ruling elites. Prior research, however, audited Yandex’s algorithms largely within Russia. In contrast, this study is the first to assess the role of Yandex’s web search algorithms as a resource for Russia’s informational influence abroad. To do so, we conduct a comparative algorithm audit of Google and Yandex in Belarus, examining the visibility and narratives of COVID-19-related conspiracy theories in their search results. By manually analysing the content of 1320 search results collected in mid-April to mid-May 2020, we find that, compared with Google, (1) Yandex retrieves significantly more conspiratorial content (2) that close to exclusively suspects US plotters to be behind the pandemic, even though the virus spread from the Chinese city of Wuhan across the globe.

Since the early 2000s, search engines have become powerful intermediaries of digital information, altering traditional news flow patterns (Wallace, 2018) and reshaping how people consume news (Fletcher and Nielsen, 2018). Aiming at assessing the sociopolitical consequences of search engine behaviour, a growing number of studies in the realm of political communication have recently implemented ‘algorithmic audits’ (Mittelstadt, 2016: 4992). For instance, researchers have analysed the diversity of search results (Puschmann, 2019; Steiner et al., 2020; Trielli and Diakopoulos, 2019; Unkel and Haim, 2019; Urman et al., 2021a), examined the presence of various biases in search results (Kravets and Toepfl, 2021; Makhortykh et al., 2022) and investigated the influence of search personalisation (Haim et al., 2018; Kliman–Silver et al., 2015). Auditing specifically the Kremlin-controlled search engine Yandex, extant research has demonstrated that the algorithms of this Russia-based intermediary are often biased toward the interests of the country’s ruling elites (Daucè and Loveluck, 2021; Kravets and Toepfl, 2021; Makhortykh et al., 2022; Wijermars, 2021). These studies, however, discussed Yandex’s algorithms and their impact solely within Russia. In contrast, none of these studies explicitly investigated the role of Yandex as a resource for Russia’s ruling elites to influence audiences abroad (for exceptions with pertaining to Yandex News algorithms, see Erbsen 2019; Erbsen and Põldre, 2022).

In order to fill in this gap, this study compares the visibility and narratives of COVID-19-related conspiracy theories (CTs) in search results that were retrieved by Yandex and its US-based counterpart, Google, from within Belarus. Belarus was selected as a country case for this study because at the time of research, it was the country where Yandex had the highest market share (20%) outside of Russia (StatCounter, 2021a). We focus on CTs as these are a widely deployed element of Russia’s foreign communication efforts (Watanabe, 2018; Yablokov, 2015). Thus, the overarching research questions of this study are: At the peak of the first wave of the COVID-19 pandemic in Belarus (from mid-April to mid-May 2020), did the Russia-owned search engine Yandex contribute more to the spread of CTs than the US-owned search engine Google? Did Yandex spread more conspiracy narratives that were biased toward the interests of Russia’s ruling elites? What role did the source websites connected with Russia’s ruling elites play in the visibility of the CTs in the search results retrieved from within Belarus?

To answer these questions, we systematically scraped results from Google’s and Yandex’s web search engines at the peak of the first wave of the COVID-19 pandemic in Belarus between 22 April and 22 May 2020 (i.e., for one month). In this period, we collected the top 20 results every three days, which resulted in 11 search rounds. In every round, we deployed three search terms that targeted CTs speculating about the origins of COVID-19 with increasing ‘specificity’ (Toepfl et al., 2022: 3): ‘coronavirus’, ‘coronavirus origin’ and ‘coronavirus biological weapon’. Although Belarus has two official languages, Russian and Belarusian, we conducted queries only in Russian, since the majority (71%) of Belarusians reportedly use Russian as their main means of communication (Belstat, 2020). The dominance of Russian as the language used for Internet search in Belarus at the time of this research was even more striking. For instance, Belarusians searched for the word ‘coronavirus’ on Yandex approximately 24,000 times more often in Russian than in Belarusian (Yandex Wordstat, 2022). Adopting this approach, we obtained a dataset of 1320 search results, of which 356 were unique. In the next step, in a manual content analysis of each search result, we determined the geographic origin of the source website and its potential connection to Russia’s ruling elites, whether undebunked CTs were present and who was suspected as the main plotter behind the outbreak of the pandemic.

We begin this article by engaging with the current state of research on Russia’s foreign communication efforts. We then continue by presenting the research on search engines and misinformation, with a focus on Yandex’s search algorithms. In the subsequent findings section, we show that, at the peak of the first wave of the coronavirus pandemic in Belarus, Yandex—compared to Google—retrieved a greater number of COVID-19-related CTs and specifically CTs suspecting plotters from the US behind the pandemic. We conclude this article by discussing how these findings advance the literature on Russia’s foreign influence campaigns, as well as, on a more general level, the existing research on how search engines contribute to the spread of mis- and disinformation.

## Russia’s informational influence abroad: Yandex as an Underresearched resource

As suggested in extant research, key resources of Russia’s informational influence abroad include Russia’s two official news broadcasters, *RT* (formerly, *Russia Today*) and *Sputnik* (Kragh and Asberg, 2017; Wagnsson, 2022), so-called ‘troll armies’ (Daucè and Loveluck, 2021), social networks (*Vkontakte* and *Odnoklassniki*; Golova, 2020), regional proxy media outlets not openly linked to Russia (Navumau, 2020) and non-governmental organisations (NGOs; Kragh and Asberg, 2017). As this overview of the literature illustrates, a broad range of resources for Russia’s foreign communication apparatus have been scrutinised. However, no study to date has considered the Kremlin-controlled search engine *Yandex* and its web search algorithms as potential instruments of the Kremlin’s foreign informational influence. As an exception regarding Yandex’s news algorithms, Erbsen and Põldre (2022) compared the Russian-language news content on Yandex’s news website in Russia, Estonia and the US. They found that Yandex’s prioritization of topics differed significantly across the three countries. For example, Yandex in Russia prioritized topics related to Ukraine, while in Estonia it focused more on local elections and social welfare, and in the US—on international relations. While the authors do not speculate why the algorithm for each country generates different content, they suggest that further research is needed to determine Yandex’s role as an ‘information tool of the Kremlin’ (p. 24) in the contexts outside of Russia. To fill this gap, this study is the first to systematically investigate how Yandex’s web search algorithms contribute to spreading pro-Kremlin content in Belarus, one of the key target countries of Russia’s foreign influence efforts (Toepfl et al., 2022).

Moreover, by choosing Belarus as a country case and Russian as the language of our audit, we broaden the scope of extant research on Russia’s foreign informational influence—which so far has focused (a) on the Kremlin’s English language endeavours at the expense of its Russian language efforts (for an exception, consider Golova, 2020) and (b) on Western democracies rather than on the states in Russia’s immediate neighborhood (for notable exceptions, consider Erbsen, 2019; Navumau, 2020; Szostek, 2018). The dearth of research on Eastern European and Eurasian countries is particularly unfortunate as ‘Russian leaders have never hidden their desire to maintain or increase their influence in the post-Soviet republics’ (Szostek, 2018: 1), even though according to official rhetoric Russia long aspired to ‘lead its neighbors through natural gravity [arising from a “very close kinship of souls”], without need for coercion’ (Szostek, 2018: 2). Russia’s foreign communication efforts targeting these countries can be thus seen as attempts to foster support for Russia’s strategic goals in these countries, without the use of military force. When Russia’s military invaded Georgia in 2008, however, then-President Dmitry Medvedev openly stated that he considered the post-Soviet republics as falling within Russia’s ‘“traditional sphere of interests” (траэиционная сфера интересов, traditsionnaya sfera interesov)’ (cited in Szostek, 2018: 2). Russia’s subsequent military invasions of Ukraine in 2014 and 2022 further underlined the willingness of the Kremlin to follow up on informational campaigns with military coercion. The case of Belarus is also especially interesting considering the current political ties of Belarus’ ruling elites in the context of Belarus’s ‘further integration’ (Navumau, 2020: 461) into Russia and, more recently, Belarus’s versatile support of Russia’s war against Ukraine (for an overview of Russia’s foreign communication efforts in Belarus, see Navumau, 2020; Szostek, 2018).

Against this backdrop, we argue in this article that the search engine Yandex needs to be understood as a key resource of Russia’s foreign propaganda apparatus. The latter is coordinated by Russia’s ruling elites, which we conceive of in this study as the autocrat Vladimir Putin and his closest allies. In the following, we shall refer to this group metaphorically also as ‘the Kremlin’. As Russia’s ruling elites have significant leverage on how Yandex’ search algorithms function (as we detail in the Context section below), they have algorithmic control over what citizens in Belarus—as well as in other countries where Yandex is popular—find when they search the Internet for information. For the Kremlin, Yandex is thus a powerful instrument of foreign informational influence.

That said, it is important to highlight that disseminating CTs, in specific, has long been one of the key strategies of Russia’s domestic and foreign propaganda (see Kragh and Asberg, 2017; Watanabe, 2018; Yablokov, 2015). As Yablokov (2015) demonstrated, to cite but one example, Russia’s key foreign communication outlet RT has long deployed CTs as ‘one element within a broader array of political strategies aimed at exposing the inequities of the political and economic [world] order’ (p. 312). Already at the time of Yablokov’s (2015) research conducted in 2013 and 2014, RT’s programmes were heavily penetrated by populist, anti-elitist, conspiratorial claims ‘aimed at uniting the imagined global community of “the people” against the dangerous “Other”, represented by the US establishment’ (p. 312). As Yablokov (2015) argued, RT swiftly deployed anti-Western CTs, amongst others, to highlight the socio-economic problems of the US, deflect criticism from Russia, and reallocate legitimacy from the US to Russia in the global arena.

## How search engines contribute to the spread of conspiracy theories

This study understands CTs as ‘narratives that embody the belief that secret and influential organisations are behind the occurrence of a particular event’ (Hussein et al., 2020: 4) without ‘credible evidence’ (Radnitz, 2021: 18) being available to the public at the time to support the claim. Based on this understanding, as suggested in previous research (e.g., Douglas et al., 2019; Hussein et al., 2020; Smallpage et al., 2020), CTs are not necessarily false. For some of these claims, evidence of their truth may be provided in the course of time. In the context of the COVID-19 pandemic, at the time of this research in April–May 2020, an academic consensus emerged that the virus was highly unlikely to have originated in a laboratory, but a minority of academics still called for further investigation of such possibility (e.g., Harrison and Sachs, 2022). Therefore, we consider the claims conspiratorial, regardless of whether they might be supported by academic consensus in the future. We consider them as conspiratorial because they suspect one or more powerful actors of secretly instigating the COVID-19 outbreak without providing credible evidence of such an allegation (Douglas et al., 2019; Radnitz, 2021).

Considering the importance of search engines in the digitalised news-gathering process (Fletcher and Nielsen, 2018; Wallace, 2018), the presence of CTs in top search results could induce the formation of conspiracy beliefs. Yet, the role of search engines in promoting CTs has received little scholarly attention (for similar claims see Bandy, 2021), with a few notable exceptions in recent years (Hussein et al., 2020; Toepfl et al., 2022, 2023; Urman et al., 2021b). For instance, Hussein et al. (2020) investigated how YouTube’s search engine promotes misinformative content. They found that the proportions of CTs present in YouTube’s search results varied between 10% and 75% depending on the specific CT searched for (‘9/11’, ‘chemtrails’, ‘flat earth’, ‘moonlanding’ and ‘vaccines’; p. 19). They also suggested that YouTube was modifying its search algorithms for some conspiratorial topics (‘vaccines’) but not for others (‘chemtrails’; p. 23).

Urman et al. (2021b) compared the presence of conspiratorial content on six conspiracy-related queries (‘flat earth’, ‘new world order’, ‘qanon’, ‘9/11’, ‘illuminati and ‘george soros’) across five search engines (Google, Bing, DuckDuckGo, Yahoo and Yandex) and three locations (two in the US and one in the UK). They found Yandex to be the search engine with the highest proportion of conspiracy-promoting content (around 65%), while Google almost did not retrieve any CT (around 1%). The shares of conspiratorial content on Bing, DuckDuckGo and Yahoo varied between 25% and 30%. Similar to Hussein et al. (2020), Urman et al. (2021b) found that the proportions of the retrieved CTs varied between queries and suggested that Google was modifying its search results to exclude conspiratorial content. They further suggested that the content differences between Google and Yandex with regard to CTs were partly due to the differences in the source types featured by the engines: while Google had the highest share of scientific sources, those were absent on Yandex. Instead, Yandex prioritised ‘conspiracy-dedicated’ (Urman et al., 2021b: 14) websites and links to social media.

Similarly, Toepfl et al. (2022)—in an analysis that drew on the codebook and broader data collection infrastructure also used for this study—compared the presence of COVID-19 CTs in Google’s search results in response to local language queries across four key target countries of Russia’s foreign communication (Belarus, Germany, Ukraine and the US) and Russia in November 2020. They found relatively low proportions of conspiratorial content present in Google’s search results, which varied between 14% in Belarus and 3% in Ukraine. They also found that in some countries, sources connected with Russia’s ruling elites contributed more (e.g., in Belarus and Germany) to the visibility of CTs than in other countries (e.g., in Ukraine and the US). In another study, Toepfl et al. (2023) examined 8800 Google results from five countries (Russia, the US, Germany, Ukraine, and Belarus) in November 2020 in response to queries on COVID-19 CTs in Russian and English. They found that while the extent of conspiratorial content was not dependent on the input language (6.9% for the Russian-language queries vs 6.6% for the English-language), the narrative was: the Russian-language queries retrieved more CTs suspecting plotters from the US to be behind the pandemic (35.5%) than the English-language queries (5.8%).

## Context: Yandex and the history of its appropriation by the Kremlin

At the time of our data collection in April–May 2020, Yandex’s web search engine was the second most popular in Belarus (with a 20% market share) after the global giant Google, which had a 78% share (StatCount, 2021a). The political appropriation of Yandex’s algorithms by Russian authorities had increased gradually over the years and culminated after Russia’s full-scale invasion of Ukraine in February 2022. In 2020, the ‘golden share’ of Yandex, which gives Russian officials de facto control over Yandex’s strategic decisions, was transferred from the state-owned *Sberbank* to the newly created *Public Interest Foundation*. The foundation was supposed to represent the state’s interests in Yandex and to have ‘the power to block transactions and temporarily remove Yandex’s management if it deems it in the national interest’ (Seddon, 2019: 2). Furthermore, at the time of our research, Yandex was intensively targeted in Russia by legal regulatory mechanisms that dictated which websites to exclude from search results (e.g., news websites not registered with the Russian state communications oversight agency, *Roskomnadzor*; Wijermars, 2021) and which information to forward to authorities (Meduza, 2020). As for indirect influence, it was generally suggested that Yandex avoided conflicts with authorities by adapting to Russia’s restricted media freedom realities so as not to jeopardise its economic profits (Daucé and Loveluck, 2021), whereas Google, in contrast, could afford not to comply (e.g., Makhortykh et al., 2022). Daucè and Loveluck (2021), in their audit of Yandex’s news aggregator, concluded that Yandex is a ‘key asset in the Russian government’s overall disciplining of the country’s media and digital public sphere’ (p. 1). After Russia’s invasion full-scale of Ukraine in February 2022, the Russian government’s control of Yandex became even more apparent. The company was broadly accused of censoring the war in Ukraine from its search results and news services (e.g., Eckel, 2022; Meduza, 2022; for similar accusations against Yandex censoring information on opposition protests in Russia, see Kravets and Toepfl, 2021; Wijermars, 2021). Similarly, searching Yandex for images for ‘Bucha’ rendered no results on the massacre of civilians by Russian troops in the Ukrainian city of Bucha that happened during the Russian invasion of Ukraine (Meduza, 2022). Many employees left the company in protest, accusing Yandex of becoming an ‘engine of propaganda’ (Eckel, 2022: para. 9) and ‘a key element in hiding information about the war’ (para. 16). In response to these accusations, Yandex sold its news service Yandex.News and infotainment channel Yandex.Zen to the state-controlled media company *VK*, which owns the social network *VK* in Russia (Meduza, 2022). As of mid-2022, other Yandex services such as its web search remained under Yandex’s control and continued to operate and claim algorithmic neutrality (e.g., Meduza, 2022).

## Developing research questions and Hypotheses

Extant research has found Yandex’s web search algorithms, compared with Google’s, to systematically favor conspiratorial content in its search results (Makhortykh et al., 2022; Urman et al., 2021b). Against this backdrop, we hypothesise that COVID-19-related CTs are more visible in Yandex’s web search results also from Belarus:

H1 At the time of our research and for the queries used, CTs were more visible to Belarusian users searching on Yandex than on Google.

Furthermore, extant research within Russia has found that Yandex prioritises Kremlin-connected sources more than does Google (Makhortykh et al., 2022). Against this backdrop, we assume that the same holds true for searches from Belarus. Furthermore, on a more general note, we presume that Russia-based Yandex will retrieve more websites based in Russia—regardless of their connections to Russia’s ruling elites—than will US-based Google, even when both platforms are accessed from within Belarus. We thus hypothesise that:

H2 For the queries used, websites (a) based in Russia and (b) connected with Russia’s ruling elites are more visible in Yandex than in Google results.

As extant research has demonstrated, Russian state-connected news websites typically convey the narratives of Russian authorities in their news stories (Watanabe, 2017). In addition, research has shown that Russia’s ruling elites regularly engage in disseminating CTs, and that these CTs often carry an anti-US stance (Kragh and Asberg, 2017; Makhortykh et al., 2022; Navumau, 2020; Watanabe, 2018; Yablokov, 2015). Against this backdrop, we examine the relationship between the sources connected with Russia’s ruling elites and the visibility of undebunked CTs and CTs blaming the US for the COVID-19 outbreak in search results in response to the three Russian-language queries. Hence, we formulate the following three research questions: At the time of our research and for the queries used,

RQ1 To what extent do (a) Russia-based websites and (b) websites connected with Russia’s ruling elites contribute to the visibility of undebunked CTs?

RQ2 Who are the (allegedly clandestine) plotters behind the COVID-19 pandemic accused in undebunked conspiracy narratives retrieved by (a) Google and (b) Yandex?

RQ3 Who are the (allegedly clandestine) plotters behind the COVID-19 pandemic in the search results from (a) Russia-based websites and (b) websites connected with Russia’s ruling elites?

## Methods

### Data collection

Our dataset consisted of Russian-language search results from Yandex and Google in Belarus for three queries: ‘coronavirus’ (original query in Russian: коронавирус), ‘coronavirus origins’ (коронавирус происхожДение) and ‘coronavirus biological weapon’ (коронавирус биолодгическое оружие). We chose the queries based on their gradually increasing specificity to the topic of our interest: CTs speculating about the origins of the COVID-19 pandemic. We fielded our queries in real time using anonymous HTTP GET requests (no browser history, no cookies, and no logged-in user) via the *requests* library in Python. All queries were routed through rotating proxy servers in Belarus, meaning that every search was executed through a different proxy (i.e., a different Belarusian IP address). We used multiple proxies to reduce spatial bias (Ballatore, 2015), ensure that our data was representative of Belarus in general (as opposed to a single location within Belarus) and manage Yandex’s aggressive bot detection mechanisms, which regularly blocked IPs after only a single automated query. We regularly checked whether the IPs of our proxies were identified as being in Belarus. For the default browser (included in the User Agent string of the requests), we chose the most widely used browser in Belarus, Chrome (68% market share, StatCounter, 2021b). It is generally suggested that search engine users rarely go beyond the first page (i.e., approximately top 10) of search results (e.g., Urman et al., 2021a). In this study, however, we opted to examine the top 20 search results. We did so because we assumed that in the exceptional situation of a massive global health crisis, and particularly when searching for conspiracy narratives that were allegedly hidden from the public by powerful actors, users were likely to go beyond the first page of search results.

We collected data for one month (22 April–22 May 2020). We opted for this time frame as it covers the peak of the first wave of the coronavirus pandemic in Belarus. In this period, we conducted 11 search rounds in three-day intervals, using the same queries. By doing so, we aimed to mitigate any undue influence of search randomisation, which was shown to affect search results particularly on Yandex (Makhortykh et al., 2020), and the specific day on which the search was conducted (temporal bias, see Ballatore, 2015). Overall, our dataset consisted of 1320 search results (660 for Yandex and 660 for Google), of which 356 search results were unique (i.e., linked to different URLs).

### Data analysis

The webpages to which the unique search results linked were then subjected to manual content analysis (*N* = 356). The coding was implemented by two co-authors of this article who are native or fluent speakers of Russian and Belarusian and familiar with the media landscapes of these countries. Before starting the coding effort, intercoder reliability tests on 80 items yielded satisfactory results. Among others, we coded whether an (undebunked) CT was mentioned by the webpage (Krippendorff’s α = 0.862) and whether the website was connected with Russia’s ruling elites (α = 0.915). Only sources with explicit connection to Russia’s ruling elites (e.g., direct or indirect state ownership and ownership by regime-friendly media moguls or their [ex-]spouses) were coded as such. Examples of such in our dataset include the websites of the Russian oligarch-owned news agency *RBC* (rbc.ru) and state-owned news agency *RIA Novosti* (ria.ru). Furthermore, we identified the geographic origin of the source webpages (α = 0.832) and the geographic origin of the main plotter suspected in each search result with an undebunked conspiracy narrative (α = 0.723). Examples of websites based in Russia, but not connected with Russia’s ruling elites in our dataset include the opposition media outlets *Novaya Gazeta* (novayagazeta.ru) and *Mediazona* (zona.media), as well as multiple commercial COVID-19 case trackers (e.g., coronavirus-monitor.ru, koronavirus-today.ru). For more details on the coding procedure, see the Online Supplementary File. All proportions reported in the Findings section were calculated for each search term on each day. We then calculated means across the three search terms for each day and, finally, computed means and medians across the 11 search rounds.

## Findings

Of 356 unique search results across both search engines, we identified (1) 178 that did not mention CTs, (2) 89 that mentioned CTs but debunked them and (3) 71 that mentioned at least one undebunked CT. Inaccessible websites (*N* = 18) were excluded from the analysis (5 for Google and 13 for Yandex).

### Yandex retrieves more than two times more undebunked CTs than Google

H1 posited that at the time of our research and for the queries used, CTs are more visible to users searching on Yandex than on Google. The search results on Yandex were more than two times more likely (*M* = 31.61%, *Mdn* = 30.44%) to feature undebunked CTs than search results on Google (*M* = 13.90%, *Mdn* = 11.67%). To test these differences, we conducted a non-parametric Mann–Whitney U test for independent samples used when the assumption of the within-group independence of the observations was violated (since searches performed on one search engine at different times were correlated with each other) and the sample sizes were small (*N* = 11 search rounds per search engine). The test indicated significant differences between the groups (*U* = 0.0, *p* < 0.001). Thus, H1 is strongly supported.

### Yandex retrieves more Russia-based and Kremlin-connected websites than Google

**Figure 1.**
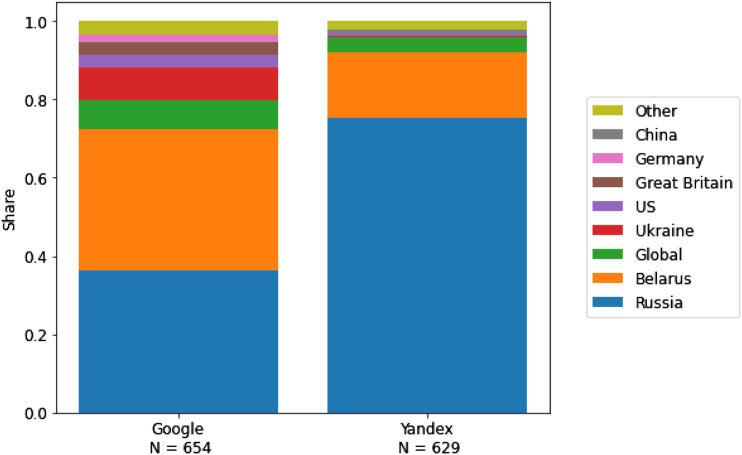
Geographic origins of sources of search results on google and yandex. *Note.* Inaccessible websites were excluded from this analysis.

H2b hypothesised that websites (a) based in Russia and (b) connected with Russia’s ruling elites are more visible on Yandex than on Google. Figure 1 illustrates the geographic origins of the sources of the search results for Google and Yandex. A Mann**–**Whitney U test indicated that Russia-based websites were more present on Yandex (*M* = 75.36%, *Mdn* = 75.93%) than on Google (*M* = 36.28%, *Mdn* = 31.84%), *U* = 0.0, *p* < 0.001. Thus, our data supports H2a. An additional exploratory analysis of the most frequent Russia-based websites in our dataset revealed that, in contrast to Google, Yandex retrieved not a single Russia-based non-Kremlin-connected mass media news website in its search results.

Next, we tested whether the share of search results from sources connected with Russia’s ruling elites was significantly greater for Yandex (*M* = 39.81%, *Mdn* = 39.16%) than for Google (*M* = 30.77%, *Mdn* = 28.33%), *U* = 11.0, *p* = 0.001. While our data supports H2b, it may be argued that the difference of 9.04% points is not substantial. However, put differently, in the first twenty results, Yandex retrieved on average eight Kremlin-connected websites, whereas Google retrieved only six.

### How Russia-based and Kremlin-connected websites contributed to the visibility of CTs

RQ1 asks how (a) Russia-based and (b) Kremlin-connected websites contributed to the visibility of undebunked CTs in the search results of Google and Yandex. Overall, our results demonstrated that approximately two thirds of all undebunked CTs retrieved by both search engines came from Kremlin-connected websites. The proportions of Kremlin-connected websites responsible for spreading undebunked CTs were approximately equal on Yandex (*M* = 71.61%, *Mdn* = 73.21%) and Google (*M* = 65.86%, *Mdn* = 53.33%), *U* = 42, *p* = 0.24. Across all results, we found that Russia-based websites (*M* = 87.45%, *Mdn* = 87.3%) were approximately seven times more likely to mention undebunked CTs than their non-Russia-based counterparts (*M* = 12.55%, *Mdn* = 12.7%), *U* = 121, *p* < 0.001. Similarly, Kremlin-connected websites (*M* = 71.45%, *Mdn* = 73.21%) were more than two times more likely to mention undebunked CTs than their non-Kremlin-aligned counterparts (*M* = 28.55%, *Mdn* = 26.79%), *U* = 120, *p* < 0.001.

### Main plotters Behind the COVID-19 pandemic on Google and Yandex

**Figure 2.**
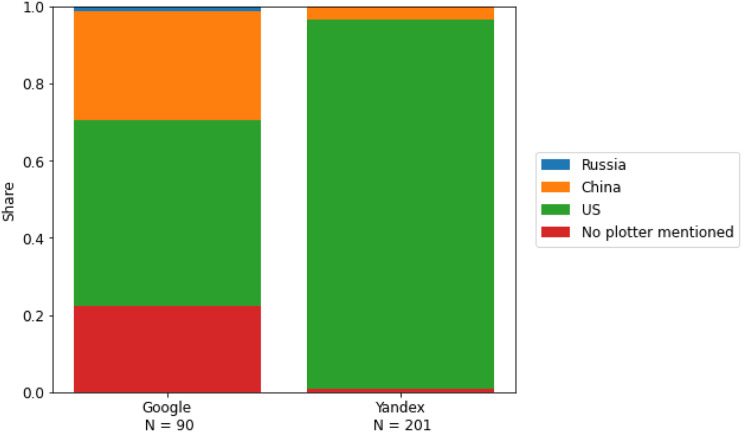
Geographic origins of the main plotters mentioned in the search results with undebunked conspiracy theories.

RQ2 asks about the geographic origin of the main plotters responsible for the outbreak of the COVID-19 pandemic mentioned in the search results. Overall, 67 (75.3%) of our search results containing undebunked CTs on Google and 200 (99.5%) on Yandex mentioned a specific plotter. As Figure 2 demonstrates, search results with conspiratorial content on Yandex close to exclusively (95.7%) suspected plotters from the US, and only 3.5% suspected plotters from China. Notably, not a single CT on Yandex suspected plotters from Russia. One example of a CT narrative retrieved from the Yandex results that blamed the US was an article from the conservative television channel *Tsargrad* (Yegorova, 2020) sponsored by the Kremlin-affiliated oligarch Konstantin Malafeev. The article states that the US was ‘caught red-handed’ (Yegorova, 2020) in bringing COVID-19 to China on purpose, and three Russian experts confirmed it. The first of the experts, a Russian academic, claimed that COVID-19 was developed by the US as a biological weapon to reduce the planet’s population and targeted primarily Asian countries such as China. Another expert, a former inspector of the United Nations Commission on Chemical, Bacteriological and Biological Weapons, allegedly supported these claims, naming COVID-19 a product of US laboratories created as an ‘ethnically specific biological weapon’ (Yegorova, 2020) to win the economic war with China. One ‘proof’ of the US guilt that he presented is that the US and the UK produced the vaccine against the virus in a very short time, which is deemed impossible. Finally, the third expert, introduced as a military observer, supported this claim by pointing out that the US has 400 bio laboratories across the world, 40 of which are at the Russian borders, and that COVID-19 is ‘the new way of establishing the supremacy of the US over its main antagonists’ (Yegorova, 2020).

### Russia-based and Kremlin-connected websites blame the US for the COVID-19 outbreak

Next, we analysed the geographic origin of the main plotters mentioned in the search results with undebunked CTs from (a) Russia-based websites and (b) websites connected with Russia’s ruling elites (RQ3). Overall, 237 (93%) search results from Russia-based websites and 189 (93%) from websites connected with Russia’s ruling elites mentioned a specific plotter. Close to exclusively, these blamed plotters from the US (93.7% and 97.4%, respectively), with the only other plotters mentioned being plotters from China (6.3% and 2.6%, respectively). Not a single search result from Russia-based or Kremlin-controlled sources in our dataset suspected plotters from Russia.

Across all the search results, we found that Russia-based websites were approximately 10 times more likely (*M* = 90.43%, *Mdn* = 92.11%) to mention undebunked CTs that blamed US plotters than their non-Russia-based counterparts *(M* = 9.32%, *Mdn* = 7.89%), *U* = 121, *p* < 0.001. Similarly, Kremlin-aligned websites were around three times more likely (*M* = 74.06%, *Mdn* = 71.67%) to blame US plotters for the COVID-19 outbreak than non-Kremlin-aligned websites (*M* = 25.69%, *Mdn* = 28.33%), *U* = 121, *p* < 0.001.

## Discussion

Since the 2000s, search engines have become indispensable intermediaries for selecting, ranking and redistributing political information online (Puschmann, 2019; Steiner et al., 2020; Wallace, 2018). This study audited the web search algorithms of Google and Yandex for spreading CTs regarding the origins of the COVID-19 virus based on three search terms with increasing specificity (‘coronavirus’, ‘coronavirus origins’ and ‘coronavirus biological weapon’). Using data from 1320 search results, we found that (1) Yandex was more than two times more likely to feature undebunked CTs in its search results than Google, (2) the majority of CTs both on Google and Yandex came from Russia-based websites and websites connected with Russia’s ruling elites and (3) conspiratorial content retrieved in searches on the Kremlin-controlled search engine Yandex close to exclusively suspected plotters from the US behind the outbreak of the pandemic, even though the virus was first observed several weeks before in the Chinese province of Wuhan.

### How Yandex contributes to the spread of conspiracy theories

Extant research has suggested that Yandex’s algorithms are manipulated toward pro-Kremlin narratives of Russia’s ruling elites (Daucè and Loveluck, 2021; Kravets and Toepfl, 2021; Makhortykh et al., 2022; Wijermars, 2021). These studies, however, audited Yandex’s algorithms and their impact solely within Russia, leaving out Yandex’s potential for mediating the communication efforts of Russia’s ruling elites outside Russia. The results of this study demonstrated that the findings of prior research on Yandex for Russia are also valid for Belarus and Yandex’s web search algorithms show similar bias toward Kremlin-connected websites and pro-Kremlin narratives also if accessed from abroad. Specifically, we found that compared to Google, Yandex in Belarus retrieved larger proportions of websites connected with Russia’s ruling elites (see H2). These Kremlin-connected websites were more likely to contain undebunked CTs than their non-Kremlin-connected counterparts (see RQ1) and were responsible for the majority of undebunked conspiratorial content not only on Yandex but also on Google.

In addition, our findings demonstrated profound differences with regard to the prioritisation of conspiratorial content between Yandex and Google: Yandex’s web search algorithms were more than two times more likely to retrieve undebunked CTs in its search results than Google’s (H1). While extant research has typically suggested that the content differences between Google and Yandex with regard to CTs are mostly due to the differences in the prioritised source types (Urman et al., 2021b), moderation policies (Hussein et al., 2020; Urman et al., 2021b) or political alignment of the sources of the search results (Makhortykh et al., 2022), this study was the first to consider the link between the geographic origin of the sources of search results (Russia-based vs. non-Russia-based) and the extent of the conspiratorial content present in the search results. In the context of this study, we found that the geographic origin of the search results played a major role (RQ1/RQ3). In the scrutinised Belarusian context, the geographic origin of the search results made a difference not only with regard to how many undebunked CTs were spread, but also with regard to the attribution of the blame. Specifically, we found that Russia-based websites were much more likely to contain undebunked CTs and to blame US plotters for the pandemic than their non-Russia-based counterparts. The findings of this study thus suggest that Yandex’s display of more conspiratorial content in its search results compared to Google is due to Yandex’s prioritisation of (a) Russia-based and (b) Kremlin-connected websites, which are more likely than their non-Russia-based and non-Kremlin-connected counterparts to contain undebunked CTs.

At the most abstract level, even though Yandex has repeatedly claimed that its search algorithms are objective and based solely on algorithmic decision-making (e.g., Meduza, 2022), our findings can be interpreted as further de-legitimising Yandex’s claims of political neutrality—not only with regard to how its algorithms function within Russia (see Daucè and Loveluck, 2021; Kravets and Toepfl, 2021; Makhortykh et al., 2022) but also with regard to how they function abroad. In addition, our findings demonstrated the possibility of profound information inequalities forming between the users of Google and Yandex in Belarus. Yandex, by favoring CTs and Kremlin-connected websites in its search results, formed an information ecosystem for its users parallel to that of Google. Thus, people informing themselves about the origins of the COVID-19 virus exclusively on Google or Yandex were likely to receive drastically different pictures after using the respective search engine.

### Yandex as Russia’s resource for informational influence in Belarus

Prior research has considered a broad range of Russia’s resources for informational influence abroad (see Daucè and Loveluck, 2021; Golova, 2020; Wagnsson, 2022). However, this study is the first to consider web search engine operation or, as is the case with Yandex in Russia, the appropriation of a web search engine’s algorithms, as a proactive strategy of Russia’s government to propagate its agenda and perspectives on the world abroad. Our findings vividly demonstrate the success of these endeavors: at the time of our research and for the queries used, Yandex prioritised the messages of Russia’s state-controlled media in the Belarusian context. Hereby, it contributed to the spread of CTs and pro-Kremlin narratives (i.e., CTs with an anti-US stance) among the Belarusian audience. Extant research has claimed that CTs are used by Russia’s ruling elites as a ‘political instrument’ (Yablokov, 2015: 301; Kragh and Asberg, 2017; Watanabe, 2017) to achieve their foreign communication ventures. In line with this, this study found that almost all the Kremlin-aligned websites with undebunked CTs in our dataset featured a specific type of plotter, that is, plotters from the US, behind the outbreak, even though the virus has spread from the Chinese city of Wuhan across the globe (RQ3). This again highlights the conspiratorial focus of these media and their focus on building a so-called ‘us against the others’ mindset with a specific ‘other’ in mind: the US (Kragh and Asberg, 2017; Yablokov, 2015).

As these findings illustrate, Yandex’s prioritisation of Russia-based and Kremlin-connected sources massively increased the visibility of anti-US CTs for Belarusian Internet users. Against this backdrop, the search engine Yandex needs to be understood as a key resource of Russia’s foreign propaganda apparatus, fulfilling similar purposes as Russia’s prime foreign communication outlets RT and Sputnik (Kragh and Asberg, 2017; Wagnsson, 2022) or the so-called ‘troll armies’ (Daucè and Loveluck, 2021). Similar to these entities, the algorithmic intermediary Yandex is under the tight control of Russia’s ruling elites, and it contributes to increasing the visibility among foreign audiences of pro-Kremlin content. In this sense, we believe that the conclusions of this audit about how Yandex mediated one specific disinformation issue (COVID-19 CTs) can be extrapolated to the broadest range of political and cultural messages that touch upon the Kremlin’s foreign policy interests. Moreover, in our view, they are also broadly generalisable to other populations and countries, where Yandex web search still has relatively substantial market shares. At the time of this research in April-May 2020, these contexts included Uzbekistan (16% market share), Tajikistan (15%), Turkmenistan (15%), Kazakhstan (14%) and Kyrgyzstan (9%; StatCounter, 2021a).

Our findings also raise more general concerns about information sovereignty of Russia’s neighbor states. In our case study of Belarus, we found that Yandex largely forwarded its Belarusian audience to Kremlin-connected sources and pro-Kremlin (i.e., anti-US) conspiracy narratives. This is problematic, for example, in the context of Russia’s recent full-scale invasion of Ukraine, since our findings suggest that Belarusian users accessing information via Yandex might be informed only of Russia’s ruling elite’s perspective on the war, which is framed as a ‘special operation’ to free the people of Donbass rather than a ‘war’ (Eckel, 2022). Since Yandex is censoring information on the war in Ukraine (Eckel, 2022; Meduza, 2022), news, for example, about civilian casualties among the Ukrainian population, Russia’s military losses and critical information about Russia’s actions will frequently not even reach Belarusians who rely on Yandex as an entry point to the web.

## Limitations and promising paths for future research

This study has several limitations, which, however, open promising paths for future research. First, this study has accomplished a comparative algorithm audit of the web search algorithms of Google and Yandex in Belarus based on only one issue: CTs speculating about the origins of the COVID-19 pandemic. It appears highly plausible that our key finding that Yandex results retrieved in Russian from outside Russia are biased towards the interests of the Kremlin is broadly generalisable to a broad range of political issues. However, in order to substantiate this claim, future research needs to analyse a larger number of political issues, including a larger set of CTs or politically controversial topics. Furthermore, future research can compare the results retrieved by Yandex in Belarus to those retrieved in other countries where Yandex has relatively substantial market shares or even to those retrieved from within Russia.

Second, we employed reverse engineering to perform our algorithmic audit of Google and Yandex web search algorithms. Based on our approach, we could not investigate the rationales that motivated the designs of the two algorithms, the extent of the actual pressure by the Belarusian and Russian authorities on Yandex specifically with regard to how its algorithms operate in Belarus, and the specific rules that were incorporated in the Yandex algorithms that resulted in the dominance of the Russia-based and Kremlin-connected sources. Future research might complement our findings, for example, by conducting interviews with media experts, (former) government officials and (former) employees of the company to gain more qualitative insights. Particularly intriguing questions for future research, considering also the consequences of the split of the company after Russia’s invasion of Ukraine in 2022, include how important the international dimension is for Yandex, how the control structures in different countries are and will be organised, how Yandex distributes tasks across its branch offices in different geographic locations, how political attitudes of Yandex-users differ from those of non-Yandex users across Eurasia, and why Yandex-users choose using Yandex over Google in the first place. By following up on these paths of scrutiny, future research could shed more light on the complex mechanisms of the mediation by Yandex’s algorithms of the messages of Russia’s ruling elites abroad. Particularly in light of Russia’s war against Ukraine, it is crucial to continue investigating Russia’s tools for informational warfare.
